# Bacteria and associated antibiotic resistance in air filter-derived biological material from utility vehicles at mechanical-biological treatment plants

**DOI:** 10.13075/ijomeh.1896.02785

**Published:** 2026

**Authors:** Anna Kozajda, Emilia Miśkiewicz, Jan Gawor, Robert Gromadka

**Affiliations:** 1 Nofer Institute of Occupational Medicine, Biological Safety Unit, Łódź, Poland; 2 Polish Academy of Sciences, DNA Sequencing and Synthesis Facility, Institute of Biochemistry and Biophysics, Warsaw, Poland

**Keywords:** occupational exposure, bacteria, antibiotic resistance genes, occupational microbiome, occupational resistome, MBT

## Abstract

**Objectives:**

The study objective was to assess occupational exposure to bacteria and antimicrobial resistance genes (ARGs) present on air filters of utility vehicles used in the working environment of mechanical-biological treatment (MBT) facilities, in the context of workers health risks.

**Material and Methods:**

The study was conducted in summer 2024 in 9 air filters from utility vehicles used in MBT plants in Poland. External filters were removed from the vehicle's ventilation system, packed and shipped according to instructions. From the duplicate filters samples DNA was isolated and high-throughput next-generation sequencing (NGS) was carried out. Bioinformatic data analysis was conducted to detect bacteria and ARGs in air filters’ surfaces.

**Results:**

Totally, 34 bacterial taxa were detected in relative abundance ≥0.5%. The genera most frequently present at the highest relative abundances: *Saccharomonospora*, *Thermobifida*, *Nocardiopsis*, *Pectobacterium*, *Aerococcus*, *Thermoactinomyces*, *Novibacillus* and *Streptomyces*. Across all bacteria isolated from the analyzed filters, regardless of their relative abundance, a total of 91 taxa were classified into risk groups 2 or 3 (86 and 5 taxa, respectively). The most frequently detected ARGs were those encoding resistance to a single class of antibiotics (*AAC(3)-VIIa*, *aadA2*, *ANT(6)-Ia*, *APH(3’’)-Ia*, *APH(3’’)-Ib*, *APH(6)-Id*, *cml*, *cmx*, *lnuA*, *lnuD*, *novA*, *parY*, *sul2*, *vanHF*, *vanJ*, *vanRA*, *vanRI*, *vanRO* – each at least on 4 air filters). Antimicrobial resistance genes encoding multi antibiotic resistance were also detected: CRP, *emtA*, *erm(34)*, *erm(36)*, *ermA*, *ermC*, *ermF*, *ermG*, *ermT*, *ermX*, *ernB*, H-NS, *mel*, *msrA*, *msrE*, *mtrA*, *optrA*, *ramA*, *ykkD* – each at least on 1 air filter.

**Conclusions:**

Despite the limited number of analyzed filters, the study demonstrated a high bacterial species diversity in the MBT plant environment. The MBT workers are exposed to bacteria with high pathogenic potential and to ARGs encoding resistance to antibiotics used exclusively in human medicine, used in human and veterinary medicine, and not intended for human use.

## Highlights

- The mechanical-biological treatment environment is microbiologically contaminated.
- Workers are exposed to pathogenic bacteria and antibiotic resistance genes.
- The use of next-generation sequencing allowed for the detection of antimicrobial resistance genes within the air filters.

## INTRODUCTION

A resident of the EU generates on average approximately half a tonne of municipal waste per year [1]. According to data from the Central Statistical Office, in Poland in 2024 the amount of mixed municipal waste per capita reached 179 kg [2]. Municipal waste refers to waste generated in households and waste originating from other waste producers which, due to their nature and composition, are similar to household waste [3]. In Poland, pursuant to the Waste Act [3] and the Act on Maintaining Cleanliness and Order in Municipalities [4], municipal waste is subject to mandatory segregation. The waste fraction remaining after segregation is unsorted (mixed) municipal waste classified in the Waste Catalogue under the code 20 03 01 [5]. Entities responsible for collecting municipal waste are obliged to transfer unsorted waste to facilities that ensure its disposal through a mechanical–biological treatment (MBT) process. It is a complex waste-processing procedure aimed at the recovery of secondary raw materials through mechanical operations (including shredding, screening, sorting, and the separation of ferrous and non-ferrous metals) as well as the extraction of biodegradable fractions (composting or stabilisation) [6,7]. In Poland, the operation of MBT facilities is regulated by the Regulation of the Minister of Climate and Environment on the mechanical-biological treatment of unsorted (mixed) municipal waste [8]. Waste processed in these facilities constitutes both a source of and a substrate for microorganisms. Bacteria, fungi, viruses, and endoparasites of human, animal, and environmental origin may be present in such waste. Currently, expert attention is focused on bacteria due to the high probability of antibiotic resistance transmission and the resulting risk to public health [9].

The source of bacteria in the mixed municipal waste fraction is the organic fraction, residues of food products (primarily of animal origin: fats, bones, meat), human excreta (used disposable nappies/diapers), and animal excreta (mainly from dogs) [6,10–13]. The quantitative and qualitative composition of biological agents varies depending on the waste stream. The taxa of bacteria present in the working environment of MBT facilities depend largely on the morphology of the waste, climatic conditions (temperature, humidity), and the duration of waste storage before delivery to the processing plant, with no legal regulations governing these aspects [14,15].

Over the past 2 decades, the growing global problem of bacterial antimicrobial resistance has attracted increasing attention in the context of public health. Potentially pathogenic bacteria that may be present in MBT facilities are typically faecal bacteria originating from humans and domestic animals. A high prevalence of antimicrobial resistance genes is currently observed in both humans and animals, which results from the overuse of antibiotics in medicine and veterinary practice [16]. The transmission of bacteria in the MBT working environment occurs via airborne routes (through bioaerosols), direct contact (through handling waste), and indirect routes (through contaminated surfaces). Consequently, infection may occur via inhalation, through the skin (intact or damaged), through mucous membranes, and through the eyes. In occupational hygiene, the respiratory route is considered the greatest health and safety risk with respect to harmful biological agents [17,18].

The aim of the presented study was to assess occupational exposure to the taxa of bacteria, as well as antimicrobial resistance genes, present on air filters of utility vehicles used in the working environment of MBT facilities, in the context of health risks to the employees of these plants. Air filters were used as research material because they operate for up to 1000 working hours (information obtained directly from the MBT facility), during which time organic dust containing the genetic material of microorganisms accumulates in the filtering medium. This approach enables an assessment of exposure to bacteria present in workplace air over a longer time interval than that provided by a single collection of the bioaerosol sample. To characterize both the air microbiome and the resistome, next-generation sequencing (Illumina, San Diego, USA) was applied to DNA extracted from the filter-associated organic dust. The study presents the results of the first investigation of bacteria and antimicrobial resistance genes found in organic dust gathered on the filters of utility vehicles in MBT facilities in Poland.

## MATERIAL AND METHODS

### Field study

The field study was conducted in the summer season (July–August) of 2024 in Poland. The MBT facilities approached for permission to participate in the study were selected regionally so that their locations would represent the entire country. For this purpose, the nationwide Internet Waste Data Database [19] was used, which contains, among other information, data on all MBT facilities in Poland, organised by region and with the option to generate a map of their geographical locations. All facilities participating in the study required the research team to sign a confidentiality clause, including confidentiality regarding their regional locations. Therefore, the present analysis does not specify the voivodeships involved.

The process of obtaining consent began with sending formal letters to the selected facilities; however, it became evident that the time required for such a letter to be processed by an MBT facility exceeded 2 weeks and in some cases even a month. Consequently, the procedure was changed so that the formal letter was preceded by a telephone call in which the objectives and methodology of the study were presented, and the facility representative was encouraged to grant consent for participation. In nearly every MBT facility, several such conversations had to be conducted with successive representatives, including the company president and/or board members, as well as with the occupational health and safety service. Although this approach was highly demanding for the research team, it simultaneously enabled the acquisition of valuable first-hand information regarding the handling of air filters supplying air to the drivers’ cabins of utility vehicles used for waste transshipment in MBT facilities. These findings are described in the Results section.

### Material

The research material consisted of organic dust collected from 9 HEPA air filters used in loader type utility vehicles used for waste transshipment at 9 MBT facilities. In each plant there were treated unsorted municipal waste originated mainly form households, but not exclusively, also from enterprises, different services (including “beauty” branch), healthcare (including hospitals), veterinary etc. In some cases, MBT facilities were a part of larger waste processing companies and do not have access to detailed data related to waste sources. In addition, some MBT facilities processed waste collected by other companies (the waste collector differs from the operator of the MBT installation). In the ventilation systems of utility vehicles (loaders), 2 air filters are installed. The first, external filter (HEPA-type), captures bioaerosol from the air in the working hall, while the second, internal filter further purifies the air supplied to the operators cabin (remove organic dust passed through the first filter). Therefore, to detect bacteria in dust originating from the working hall, the authors selected the first (external) filter for analysis. Each filter was removed from the vehicle's ventilation system and prepared for shipment in accordance with detailed instructions provided by the research team. A uniform method of packaging and shipping the filters was established to prevent loss of dust from the filtering fabric during transport, to limit the proliferation of microorganisms, and to ensure that the material reached the laboratory as quickly as possible. Therefore, both the packaging procedure and the method of shipment were agreed in advance with the laboratory performing the molecular analyses.

Each MBT facility received from the research team a package containing new food-grade stretch film (to ensure microbiological cleanliness), 2 reinforced red plastic bags (intended for transporting infectious waste), adhesive tape, a new cardboard box, and a prepaid courier label addressed to the laboratory conducting the analysis. Immediately after removal from the vehicle, each filter was tightly wrapped in stretch film, then placed in 2 plastic bags (each individually sealed with adhesive tape), and subsequently packed into the cardboard box and dispatched without delay via courier service to the laboratory.

Additionally, a new, unused HEPA air filter was purchased and delivered to the analytical laboratory for use as a reference sample (negative control) to distinguish background signals from those originating in the tested air filters.

### Laboratory analysis

Laboratory analyses were conducted by DNA Sequencing and Oligonucleotide Synthesis Laboratory, Institute of Biochemistry and Biophysics of the Polish Academy of Sciences, Warsaw, Poland.

### Sample preparation

Samples from the delivered cabin HEPA filters were collected under aseptic conditions using sterile scissors, a razor blade, and pliers. For each filter, approx. 1 cm² fragments were excised in triplicate from the central, dust-loaded portion of the filter material, avoiding the outer frame and edge regions. Each excised fragment was immediately placed into Zymo DNA/RNA Shield preservation buffer and stored at –80°C until DNA isolation to minimize contamination and degradation of genetic material. The sampled fragment size was selected to match the input capacity of the Zymo extraction kit and to ensure efficient lysis and recovery of nucleic acids from the filter material.

### DNA isolation

Isolation of DNA was performed independently in duplicate from each filter using the ZymoBIOMICS DNA Miniprep kit (Zymo Research, Irvine, USA) according to the manufacturer's protocol. The procedure included mechanical homogenisation of the filters in a lysis buffer using FastPrep-24 5G device (MP Biomedicals, Solon, USA) and the use of dedicated extraction columns. The concentration and purity of the isolated DNA were assessed by electrophoresis on a 1% agarose gel and by fluorometric measurement using a Qubit 3.0 instrument (Thermo Scientific, Tewksbury, USA) with DNA High Sensitivity kit reagents (Thermo Scientific, Tewksbury, USA).

### High-throughput next-generation sequencing

High-throughput next-generation sequencing (NGS) was then performed to enable taxonomic characterization of the bacterial communities present in the samples and to detect antibiotic resistance genes. For each filter, 2 independent sequencing libraries were prepared, with each library constructed from 1 of the 2 independently obtained DNA isolates. DNA library preparation and amplification were performed using the high-fidelity KAPA HiFi Polymerase (Roche, Basel, Switzerland) and the QIAseq Ultralow Input Library KitQiagen, Hilden, Germany) following the manufacturer's instructions. Sequencing was carried out on a NovaSeq 6000 (Illumina, San Diego, USA) instrument in paired-end mode (2×150 bp) at the Institute of Biochemistry and Biophysics of the Polish Academy of Sciences. Two types of controls were included in the analysis:

– a negative control of the library preparation (potential laboratory background), processed and sequenced alongside all samples;
– a DNA library constructed from material isolated from a new, unused HEPA filter, serving as an additional control of the overall method. These controls were used to monitor potential contamination and to distinguish true environmental signals from background noise.

### Bioinformatic analysis of results

Bioinformatic analyses were performed in collaboration with the sequencing team using standardized pipelines in a Linux environment. Low-quality reads were removed with fastp v. 0.23.2 [20]. Technical replicates were merged for each sampling site before downstream analyses. To reduce non-target and background contamination, reads were further processed with HoCoRT v. 1.1.0, and sequences matching the human genome, the PhiX control genome, and the clean-filter negative control were removed. Inclusion of the clean-filter control enabled identification of background or reagent-derived contaminants and improved the accuracy of metagenomic profiling, particularly for low-biomass samples.

Taxonomic classification was performed with Kraken2 v. 2.0.8 [21] using the k2_pluspfp_20240605 database, which includes reference sequences for bacteria, archaea, fungi, viruses, plants, and protists. The results were refined with Bracken v. 2.9 to estimate the relative abundance of identified microorganisms. Taxonomic count tables were then processed in R v. 4.2.3. Analyses were restricted to bacteria, and species-level analyses included only taxa assigned to the species rank. For each sample, species counts were normalized to the total number of bacterial reads to obtain relative abundance within the bacterial fraction.

Alpha diversity was assessed using 4 indices:

– observed richness – reflecting the number of detected species,
– Chao1 – estimating total richness while accounting for rare taxa,
– Shannon diversity – reflecting both richness and evenness,
– Simpson diversity – emphasizing dominance and community evenness.

Species composition was also visualized as a heatmap using ComplexHeatmap v. 2.18.0. Only taxa reaching ≥0.5% relative abundance in ≥1 sample were shown individually, whereas the remaining low-abundance taxa were grouped as “Others.”

In parallel, reads were assembled into contigs using metaSPAdes v. 3.14.1 [22] with default parameters. Antibiotic resistance genes were identified with Abricate v. 1.0.1 using the CARD database [23], and AMR profiles were visualized as heatmaps in R with ComplexHeatmap v. 2.18.0.

Only bacteria posing a health risk to exposed individuals, that are classified, in accordance with Polish and European Union legislation, into hazard groups 2 and 3 are presented regardless of their percentage share in the analysed material. In this case, the ≥0.5% threshold described above was waived due to their pathogenicity. The authors adopted the assumption that exposure to even a low concentration of a pathogen may result in infection in susceptible individuals, including those with reduced immunity (even temporarily).

## RESULTS

### Bacterial taxa

In total, across all analysed air filters, 34 bacterial species met the relative abundance threshold of ≥0.5% established for this analysis.

Table 1 presents information on the time of filter submission for analysis, the processing capacity of the MBT installation in which the utility vehicle equipped with the analysed air filter was used, and the operating duration of that filter. The examined air filters originated from 9 loader-type utility vehicles used in MBT installations with varying annual processing capacities, ranging from 3630 tonnes (F6) to 210 000 tonnes (F2). The operating time of the filter, expressed in machine hours, was reported by 6 out of 9 facilities; the shortest duration was noted for F3 (205 operational hours), whereas F2 and F6 were used the longest (1000 operational hours each). Unfortunately, the analysis of data incorporating these variables did not reveal any relationships, preventing inference regarding their impact on the obtained results.

**Table 1.** Processing capacity of installation, transfer and operating time of air filters in utility vehicles at the mechanical-biological treatment installations, Poland, 2024

Filter No.	Date of filter transfer to the laboratory	Annual mass of processed waste [t/year]	Filter operating time [operational h]
F1	July 23	80 000	500
F2	July 23	120 000	1000
F3	July 29	210 000	250
F4	August 07	80 000	500
F5	August 02	99 472	500
F6	July 23	3630	1000
F7	August 26	100 000	n.a.
F8	August 16	100 000	n.a.
F9	August 08	51 000	n.a.

n.a. – not available (refusal to provide information by MBT management).

The analysis of the relative abundance of bacterial species expressed as percentages, using the ≥0.5% threshold for the presence of a given species. The cumulative percentage of the relative abundance of bacteria present at ≥0.5% in the analysed sample varied across filters, from 16.9% (F4) to 63.8% (F2).

**Figure 1.**
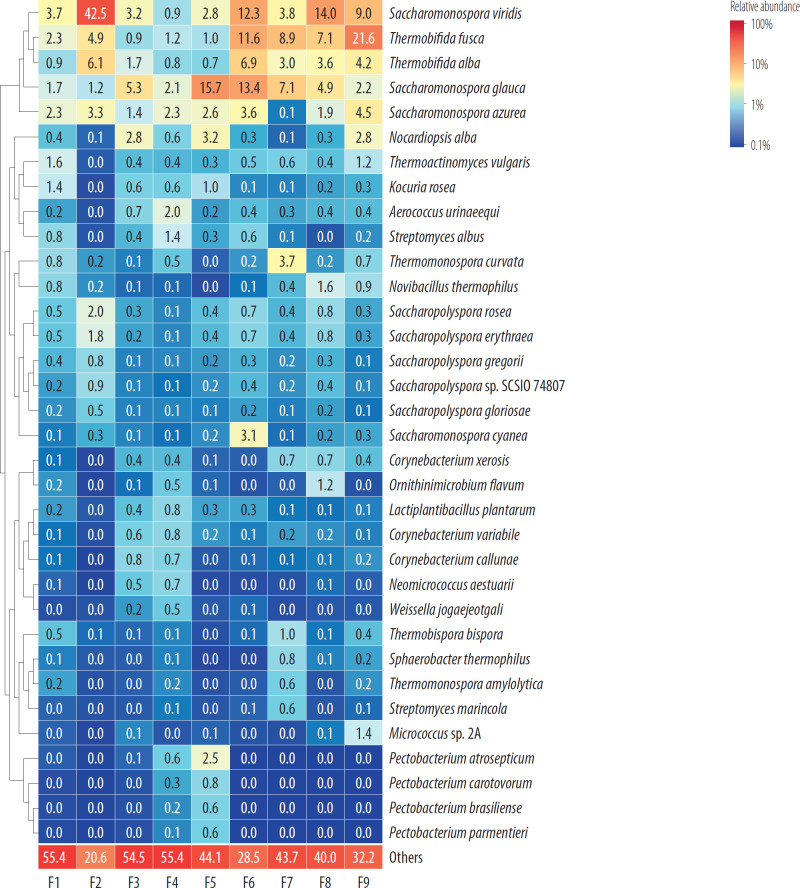
Heatmap of the most abundant bacterial species across 9 air filters in utility vehicles at the mechanical-biological treatment installations, Poland, 2024

Figure 1 presents the percentage relative abundance of bacterial species identified on each of the analysed air filters.

The heatmap shows relative abundance (%) of dominant bacterial species in samples F1–F9, normalized to total bacterial reads within each sample. Species reaching ≥0.5% relative abundance in at least 1 sample were plotted individually, while the remaining taxa were grouped as “Others.” Colors represent log-scaled relative abundance, and values are displayed as percentages within each cell.

In the organic dust collected from filter F1, 13 bacterial taxa exceeded the relative abundance threshold of ≥0.5%. These microorganisms accounted for 17.5% of the total detected bacteria. The highest percentage shares were observed for *Saccharomonospora viridis* (3.97%), *Saccharomonospora azurea* (2.3%) and *Thermobifida fusca* (2.3%).

In the organic dust from filter F2, 10 bacterial taxa species/strains exceeded the adopted relative abundance threshold. These bacteria constituted as much as 63.8% of the total isolated taxa. The highest percentage shares were recorded for *Saccharomonospora viridis* (42.5%), *Thermobifida alba* (6.1%), *Thermobifida fusca* (4.9%).

In the organic dust collected from filter F3, genetic material belonging to 11 bacterial taxa was ≥0.5% in relative abundance. These bacteria represented 18.4% of the bacterial taxa isolated from this filter. The highest percentage shares were observed for *Saccharomonospora glauca* (5.3%), *Saccharomonospora viridis* (3.2%) and *Nocardiopsis alba* (2.8%).

In the organic dust from filter F4, 17 bacterial taxa were isolated whose relative abundance reached ≥0.5%. These bacteria accounted for 16.9% of the total bacterial taxa species/strains isolated from this filter. The highest percentage shares were observed for *Saccharomonospora azurea* (2.3%), *Saccharomonospora glauca* (2.1%), *Aerococcus urinaeequi* (2.1%).

In the organic dust from filter F5, 11 bacterial taxa were isolated whose relative abundance reached ≥0.5%. These bacteria constituted 31.5% of the total bacterial taxa isolated from this filter. The highest percentage shares were recorded for *Saccharomonospora glauca* (15.7%), *Nocardiopsis alba* (3.2%), *Saccharomonospora viridis* (2.8%).

Analysis of the organic material accumulated on filter F6 revealed the presence of 10 bacterial taxa with a relative abundance of ≥0.5%. These bacteria accounted for 53.5% of the total bacterial taxa isolated from this filter. The highest percentage shares were observed for *Saccharomonospora glauca* (13.4%), and *Saccharomonospora viridis* (12.3%) and *Thermobifida fusca* (11.6%).

In the organic dust collected from filter F7, 11 bacterial taxa were isolated with relative abundance ≥0.5%. These bacteria accounted for 30.6% of the total bacterial taxa isolated from this filter. The highest percentage shares were observed for *Thermobifida fusca* (8.9%), *Saccharomonospora glauca* (7.1%), *Saccharomonospora viridis* (3.8%).

Analysis of the organic dust from filter F8 revealed the presence of 10 bacterial taxa with a relative abundance of ≥0.5%. These bacteria constituted 36.6% of the total bacterial taxa isolated from this filter. The highest percentage shares were recorded for *Saccharomonospora viridis* (14.0%), *Thermobifida fusca* (7.1%), *Saccharomonospora glauca* (4.9%).

In the organic dust collected from filter F9, 10 bacterial taxa exceeded the relative abundance threshold of ≥0.5%. These bacteria accounted for 51.5% of the total bacterial taxa isolated from this filter. The highest percentage shares were noted for *Thermobifida fusca* (21.6%), *Saccharomonospora viridis* (9.0%), *Saccharomonospora azurea* (4.5%).

In total, across all analysed air filters, 34 bacterial taxa were isolated whose relative abundance reached ≥0.5%. Among these bacteria, 4 species were present on 9 filters, another 1 species on 8 filters; 6 species were detected on 4 filters; 2 species appeared on 3 filters; 8 species were isolated from 2 filters; and as many as 13 species occurred on only a single filter. The bacterial genera most frequently present at the highest relative abundances included *Saccharomonospora*, *Thermobifida* and *Nocardiopsis*.

**Figure 2.**
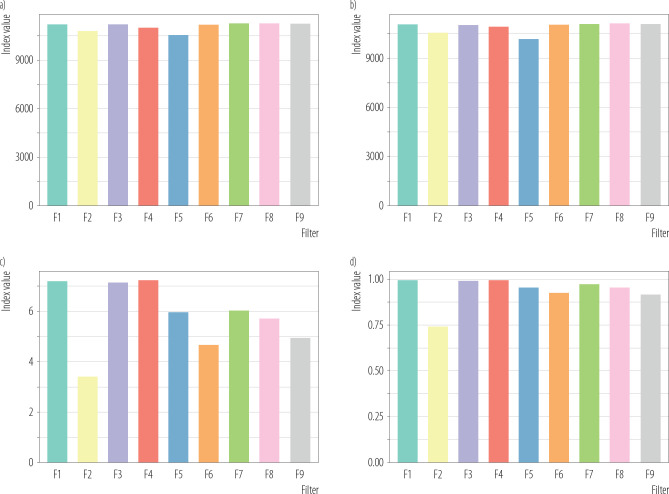
Alpha diversity of bacterial communities calculated from bacterial species-level profiles for samples F1–F9 – richness was assessed using a) Chao1 and b) observed species, while community diversity/evenness was evaluated using c) Shannon and d) Simpson indices, Poland, 2024

Alpha-diversity analysis, presented on Figure 2, indicated that bacterial richness was broadly comparable across filters, whereas diversity and evenness varied more substantially between samples. This suggests that differences among filters were driven primarily by shifts in the relative dominance of particular taxa rather than by major differences in total species richness.

Across all bacteria isolated from the organic dust collected on the analysed filters – regardless of their percentage share (relative abundance <0.5%), a total of 91 taxa classified into human risk groups 2 or 3 were identified [24]. Of these, 69 86 belonged to risk group 2, and 5 to risk group 3 (Table 2). A detailed list of pathogenic bacteria, including those belonging to risk groups 2 and 3 [24] and those classified within the ESKAPE group [25], is provided in Table 2. Notably, across all 9 filters, as many as 49 taxa classified as pathogenic to humans were detected. Furthermore, other 30 bacterial taxa were present on more than half of the analysed filters (i.e., on ≥ 5 filters).

**Table 2.** Pathogenic bacteria identified in studied filters originated from utility vehicles type used in mechanical-biological treatment installation (N = 9), Poland, summer season 2024

Bacteria^a^	Risk group^b^	Positive filters [n]
*Acinetobacter baumannii* ^c^	–	9
*Actinomadura madurae*	2	9
*Actinomyces israelii*	2	9
*Actinomyces* spp.	2	9
*Bacillus anthracis*	3	6
*Bacteroides fragilis*	2	8
*Bacteroides* spp.	2	8
*Bartonella (Rochalimaea)* spp.	2	6
*Bordetella bronchiseptica*	2	9
*Bordetella parapertussis*	2	1
*Bordetella* spp.	2	9
*Brachyspira* spp.	2	3
*Brevibacterium linens*	2	9
*Burkholderia cepacia*	2	9
*Burkholderia pseudomallei*	3	9
*Campylobacter* spp.	2	6
*Cardiobacterium hominis*	2	7
*Clostridium botulinum*	2	9
*Clostridium perfringens*	2	9
*Clostridium* spp.	2	9
*Clostridium tetani*	2	8
*Corynebacterium diphtheriae*	2	9
*Corynebacterium minutissimum*	2	8
*Corynebacterium pseudotuberculosis*	2	6
*Corynebacterium* spp.	2	9
*Corynebacterium ulcerans*	2	3
*Edwardsiella tarda*	2	9
*Ehrlichia* spp.	2	1
*Eikenella corrodens*	2	3
*Elizabethkingia meningoseptica*	2	5
*Enterobacter* spp.^c^	2	9
*Enterococcus faecium* ^c^	2	9
*Enterococcus* spp.	2	9
*Erysipelothrix rhusiopathiae*	2	6
*Escherichia coli*	2	9
*Gardnerella vaginalis*	2	1
*Haemophilus influenzae*	2	7
*Haemophilus* spp.	2	8
*Helicobacter pylori*	2	8
*Helicobacter* spp.	2	2
*Klebsiella oxytoca*	2	9
*Klebsiella pneumoniae* ^c^	2	9
*Klebsiella* spp.	2	9
*Legionella* spp.	2	8
*Leptospira* spp.	2	5
*Listeria monocytogenes*	2	9
*Mycobacterium intracellulare*	2	9
*Mycobacterium kansasii*	2	9
*Mycobacterium leprae*	3	9
*Mycobacterium malmoense*	2	9
*Mycobacterium marinum*	2	9
*Mycobacterium simiae*	2	9
*Mycobacterium tuberculosis*	3	9
*Mycobacterium ulcerans* ^**^	*3*	8
*Mycobacterium xenopi*	*2*	9
*Mycoplasma* spp.	2	1
*Neisseria meningitidis*	2	2
*Nocardia asteroides*	2	9
*Nocardia brasiliensis*	2	9
*Nocardia farcinica*	2	9
*Nocardia nova*	2	9
*Nocardia otitidiscaviarum*	2	9
*Nocardia* spp.	2	9
*Pantoea agglomerans*	2	9
*Pasteurella* spp.	2	5
*Plesiomonas shigelloides*	2	7
*Porphyromonas* spp.	2	5
*Prevotella* spp.	2	9
*Proteus mirabilis*	2	9
*Proteus penneri*	2	2
*Proteus vulgaris*	2	8
*Providencia alcalifaciens*	2	8
*Providencia rettgeri*	2	8
*Providencia* spp.	2	9
*Pseudomonas aeruginosa* ^c^	2	9
*Saccharomonospora viridis*	2	9
*Salmonella (other serotypes)*	2	9
*Shigella flexneri*	2	2
*Staphylococcus aureus* ^c^	2	9
*Streptococcus agalactiae*	2	6
*Streptococcus pneumoniae*	2	7
*Streptococcus pyogenes*	2	6
*Streptococcus* spp.	2	9
*Streptococcus suis*	2	9
*Streptomyces albus*	2	9
*Streptomyces* spp.	2	9
*Thermoactinomyces vulgaris*	2	9
*Treponema* spp.	2	6
*Trueperella pyogenes*	2	9
*Vibrio cholerae*	2	5
*Vibrio parahaemolyticus*	2	8
*Vibrio* spp.	2	9
*Yersinia pseudotuberculosis*	2	1
*Yersinia* spp.	2	7

a Bacteria present in more than half of studied filters are underlined.

b According to the Regulation of the Minister of Health of 11 December 2020 amending the regulation on harmful biological factors to health in the work environment and the protection of the health of employees occupationally exposed to these factors [23].

c Bacteria included in ESKAPE group [24].

** Agents, which may present a limited risk of infection for workers because they are not normally infectious by the airborne route (3^**^ risk group), labelling in accordance with the regulation [23].

### Antibiotic resistance genes

Sequence-based detection of resistance genes present in the dust accumulated on the air filters (Table 3) revealed the presence of 44 genes encoding resistance to 1 class of antibiotics (a total of 12 antibiotic classes); 2 genes encoding resistance to 2 classes of antibiotics; 11 genes encoding resistance to 3 classes of antibiotics; 1 gene encoding resistance to 6 classes of antibiotics; 4 genes encoding resistance to 7 classes of antibiotics; and 1 gene encoding resistance to as many as 11 classes of antibiotics. The most frequently detected genes were those encoding resistance to a single class of antibiotics, the lincosamide class (*lnuA*, detected on 8 filters), as well as genes encoding multi-antibiotic resistance to the classes of lincosamides, macrolides, and streptogramins (*ermA*, *ermB*, and *ermC*, detected on 8, 7, and 7 filters, respectively).

**Table 3.** Single and multi-antibiotics resistance genes detected in air filters from utility vehicles used in mechanical-biological treatment installations (N = 9), Poland, 2024

Gene	Filter No.								
	1	2	3	4	5	6	7	8	9
Single antibiotic resistance gene									
aminocoumarins									
*novA*	–	+	–	–	–	+	–	+	+
*Streptomyces rishiriensis parY mutant conferring resistance to aminocoumarin*	–	+	–	+	–	+	+	–	+
aminoglycosides									
*AAC(3)-VIIa*	–	+	–	–	–	+	+	+	+
*aadA2*	–	–	–	–	–	–	–	+	–
*ANT(3″)-IIa*	–	–	–	–	–	–	–	+	+
*ANT(6)-Ia*	–	+	–	–	–	+	+	+	+
*APH(2″)-If*	–	–	–	–	–	+	–	+	+
*APH(3″)-Ia*	–	+	–	–	–	+	+	+	+
*APH(3″)-Ib*	+	–	–	+	–	–	–	+	+
*APH(6)-Id*	+	–	–	+	–	–	–	+	+
diaminopyrimidines									
*dfrC*	–	–	–	+	–	–	–	–	–
*dfrD*	–	–	–	+	–	–	–	–	–
*dfrG*	–	–	–	+	–	+	–	+	–
glycopeptides									
*vanA*	+	–	–	–	–	–	+	+	–
*vanHF*	+	–	–	–	–	+	+	+	+
*vanJ*	+	+	–	–	–	+	+	+	+
*vanRA*	+	–	–	–	–	+	+	+	+
*vanRI*	–	+	–	–	–	+	+	+	–
*vanRO*	+	–	–	–	–	–	+	+	+
*vanSA*	–	–	–	–	–	+	–	–	–
*vanXF*	+	–	–	–	–	+	+	–	–
lincosamides									
*lnuA*	+	+	+	+	–	+	+	+	+
*lnuD*	+	–	–	–	–	–	+	+	+
*lnuE*	–	–	–	–	–	+	–	–	+
*lnuG*	–	–	–	–	–	–	+	+	+
macrolides									
*gimA*	–	–	–	–	–	–	+	–	–
*mphC*	–	–	+	+	–	–	–	–	–
*mphE*	–	–	–	–	–	–	–	+	+
*mphO*	–	–	–	–	–	–	–	+	–
*oleC*	–	–	–	–	–	+	+	–	–
penam/penicilins									
*CARB-14*	–	–	–	–	–	–	–	–	+
*CARB-16*	–	–	–	–	–	–	–	+	–
peptide antibiotics									
*ICR-Mo*	–	–	–	–	–	–	–	–	+
phenicols									
*catA8*	–	–	+	+	–	+	–	–	–
*cml*	–	+	–	–	–	+	+	+	+
*cmx*	+	–	–	+	–	+	–	+	–
*Lactobacillus reuteri cat-TC*	–	–	–	–	–	–	–	+	+
ansamycins (rifamycin)									
Nocardia rifampin resistant bet a subunit of RNA polymerase (rpoB2)	–	–	–	–	+	+	–	–	–
*RbpA*	–	–	–	–	–	–	+	+	+
streptogramins									
*vatH*	–	–	–	–	–	–	–	+	–
sulfonamides									
*sul1*	+	–	–	–	–	+	–	+	–
*sul2*	–	–	+	–	–	+	–	+	+
tetracyclines									
*tet(K)*	+	–	–	–	–	–	–	–	–
*tet(L)*	+	–	–	–	–	–	–	–	–
Multi-antibiotics resistance gene									
2 classes									
lincosamides, macrolides									
*emtA*	–	–	–	–	–	–	–	+	+
macrolides, penam/penicillins									
*mtrA*	+	–	–	–	–	–	+	–	–
3 classes									
aminoglycosides, phenicols, tetracyclines									
*ykkD*	+	–	–	–	–	–	–	–	–
fluoroquinolones, macrolides, penam/penicillins									
*CRP*	–	–	–	–	+	–	–	–	–
lincosamides, macrolides, streptogramins									
*erm(34)*	–	–	–	–	–	–	–	+	–
*erm(36)*	–	–	+	–	–	–	–	+	–
*ermA*	+	+	+	+	–	+	+	+	+
*ermB*	+	+	+	+	–	+	–	+	+
*ermC*	+	+	+	+	–	+	–	+	+
*ermF*	–	–	–	–	–	–	–	+	–
*ermG*	–	+	–	–	–	–	–	+	–
*ermT*	–	–	–	–	–	–	–	+	–
*ermX*	+	+	–	–	–	+	–	+	–
at least 6 classes									
cephalosporins, cephamycins, fluoroquinolones, macrolides, penam/penicillins, tetracyclines									
*H-NS*	–	–	–	–	+	–	–	–	–
lincosamides, macrolides, oxazolidinones, phenicols, pleuromutilins, streptogramins, tetracyclines									
*mel*	–	–	–	–	–	–	–	–	+
*msrA*	–	–	–	+	–	–	–	–	–
*msrE*	–	–	+	–	–	–	–	–	–
*optrA*	–	–	–	+	–	–	–	–	–
carbapenems, cephalosporins, cephamycins, fluoroquinolones, glycylcyclines, monobactams, penams/penicillins, penems, phenicols, ansamycins (rifamycin), tetracyclines, triclosan									
*ramA*	–	–	–	+	–	–	–	–	–

– Negative sample.

+ Positive sample.

Resistance genes for a single class of antibiotics detected on more than half of the analysed air filters (i.e., on ≥5 filters) encoded resistance to aminocoumarins (*parY*), aminoglycosides (*AAC(3)-VIIa*, *ANT(6)-Ia*, *APH(3”)-Ia*), glycopeptides (*vanHF*, *van*, *vanRA*), lincosamides (*lnuA*), and phenicols (*cml*).

The study revealed the presence of several different genes encoding bacterial multidrug resistance. The most frequently detected were *ermA*, *ermB*, and *ermC*, which confer simultaneous resistance to lincosamides, macrolides, and streptogramins. Genes encoding simultaneous resistance to 6 or more classes of antibiotics were detected on only a few filters. Among these, *mel*, *msrA*, *msrE*, and *optrA* which confer resistance to lincosamides, macrolides, oxazolidinones, phenicols, pleuromutilins, streptogramins, and tetracyclines, that were detected on a single filter, with the exception of *msrE*, which was found on 3 filters. A gene encoding simultaneous resistance to 11 classes of antibiotics (*ramA*, responsible for resistance to carbapenems, cephalosporins, cephamycins, fluoroquinolones, glycylcyclines, monobactams, penams, penems, phenicols, rifamycins, tetracyclines, and triclosan) was detected on only 1 filter.

Analysis of individual air filters indicates that in filters F8, F9, F6, F7, and F1, as many as 38, 30, 26, 20, and 20 resistance genes, respectively, were identified. In filters F4, F2, and F3, 16, 14, and 9 genes were detected, respectively. In contrast, filter F5 contained only 3 resistance genes.

In the analysed material, genes conferring resistance to glycopeptides, phenicols, lincosamides, macrolides, and streptogramins were detected most frequently, being present on as many as 8 filters. Resistance genes for aminocoumarins, glycopeptides, and macrolides were identified on 6 filters. In contrast, genes encoding resistance to aminoglycosides, rifamycins, and sulfonamides were found on 5 filters. The frequency of detection of genes encoding resistance to other classes of antibiotics is presented in Table 4.

## DISSCUSSION

Work at positions in municipal waste processing facilities, including MBT installations, involves exposure to biological agents. Other studies have demonstrated the presence of bacterial and fungal bioaerosols in facilities processing biodegradable waste [26]. In MBT installations, the mixed municipal waste fraction is processed, and this fraction also includes food waste. Among other materials, this fraction contains animal-derived waste (bones, meat, fat), which serves both as a substrate for microorganisms responsible for decomposition and as a source of zoonotic bacteria, particularly when such materials are raw [27]. Employees of municipal waste processing facilities may be exposed to bacteria present in the work environment via inhalation, direct contact (through intact or damaged skin and the eyes), and ingestion, this includes breathing through the mouth (resulting in swallowing bacteria suspended in dust), eating/smoking, or smoking/vaping with contaminated hands [14, 25].

According to the Internet Waste Data Database [19], >170 MBT facilities processing mixed municipal waste operate in Poland. Nevertheless, obtaining consent from 9 facilities for their participation in the study proved to be an extremely challenging task. At the begging of the study the authors obtained plenty of informal information through informal telephone conversations with employees of various MBT facilities in Poland, including both facilities that agreed to participate in the study and those that were contacted for consent but declined to take part. The individuals providing information represented different management levels (managerial and supervisory staff) as well as occupational health and safety (OHS) personnel. The presidents of many facility management boards declined to allow the participation of the facilities they oversaw. In some cases, no reason for the refusal was provided; however, many facilities, during telephone conversations, expressed concerns about the potential disclosure of irregularities in the handling of air filters (driven by the high cost of new filters), and, consequently, the possibility of inspections by authorities supervising working conditions. The authors consider the inconsistency between the visible amount of dust accumulated on the filter fabric and the number of operating hours reported by some facilities as a confirmation of these concerns. Furthermore, multiple MBT facilities reported that the filters are used for significantly longer periods than recommended by the manufacturer. This may lead to a reduction in their efficiency due to stiffening and cracking of the filter fabric, as well as proliferation of bacteria and fungi on filter fabric surface followed by aerolisation in vehicles cabin [17]. However, none of the filters under the authors’ study were cracked, but the authors cannot be sure that relative abundance of bacteria taxa is linked exclusively with bioaerosol concertation.

The information collected at the initial stage of the field study indicates that within the operational areas of MBT installations, both MBT facility employees and drivers of external delivery trucks transporting waste are present (being inside the working hall to download the waste). These waste truck drivers stand next to their vehicles during waste unloading and therefore find themselves directly in the central zone of the bioaerosol cloud (this situation was observed in person by the research team thanks to the cooperation of 1 of the MBT facilities participating in the study). MBT facility employees working in 2-shifts system carry out tasks related to the operation of machinery and loader type utility vehicles used for waste transshipment and supervise the flow of the waste stream; when needed, they also perform manual cleaning and maintenance tasks that require direct contact with the waste. The average time a utility vehicle driver spends inside the cabin is no less than 50% of the work shift. Additionally, other personnel may be present in the operational areas for shorter periods, such as OHS staff and managerial personnel, as well as inspectors (including the National Labour Inspectorate and the State Sanitary Inspectorate), and technicians servicing various types of equipment, who are usually employees of external companies.

The present study focused on the qualitative composition of the bacterial bioaerosol isolated from the air filter used to purify air supplied to the driver's cabin of a utility vehicle employed for waste transshipment at an MBT facility. As anticipated, the results confirmed high biodiversity of the bacterial microbiota present in this material. The bacteria identified in the study, before depositing onto the filtering medium, constituted components of the bioaerosol in the working hall environment. Therefore, the findings can be extrapolated to inhalation exposure among all employees present outside of vehicles cabins in these installations.

Bacterial microbiota may exert infectious, allergic, or immunomodulatory effects on exposed workers. The bacterial species isolated from the air filters may cause a wide range of health effects in individuals exposed to them. The nature of these effects depends, among other factors, on the route of exposure, the taxonomic composition of microorganisms present at a given time, the presence of chemical agents in organic dust (e.g., heavy metals, pesticides), individual susceptibility, and the immune status of the worker [28,29]. The analysed air filters revealed the presence of both pathogenic bacteria (capable of causing infections in healthy individuals) and opportunistic species. Opportunistic species pose a risk to individuals with reduced immunity, and such immunosuppression may result from concurrent bacterial or viral infections or occur in the course of chronic diseases. Low immunity may also be an individually determined, genetically conditioned trait [25]. Species such as *Haemophilus* spp., *Staphylococcus aureus*, *Klebsiella pneumoniae*, and *Mycoplasma* spp. may cause pneumonia, sinusitis, or pharyngitis. *Streptococcus* spp. and *Corynebacterium diphtheriae* may lead to local skin infections, such as abscesses. In contrast, species such as *Helicobacter pylori*, *Escherichia coli* (especially verocytotoxic or enterotoxic strains, e.g., O157:H7), *Prevotella* spp., and *Vibrio* spp. may cause gastrointestinal diseases, including gastroenteritis. Additionally, *Staphylococcus aureus* and *Streptococcus* spp. may cause ocular infections, such as bacterial conjunctivitis. Inadequate personal hygiene practices by workers may also lead to infections in other organ systems, including the urinary or genital tract, caused by organisms such as *Ureaplasma parvum* and *Prevotella* spp. [30].

In the analysed air filters, numerous bacterial species belonging to the Proteobacteria and Enterobacteriaceae groups, known for their strong pro-inflammatory potential in humans, were identified, including *Escherichia coli* (e.g., verocytotoxic or enterotoxic strains such as O157:H7), *Salmonella*, *Klebsiella*, *Yersinia*, and others [29,31]. Among the bacteria Gram-negative species were also present, characterised by their ability to produce endotoxins. Endotoxins (lipopolysaccharides – LPS) are components of the bacterial cell wall that, after cell lysis, become constituents of organic dust. The biological effects of endotoxins vary depending on the producing species; they may exert both pro-inflammatory and anti-inflammatory effects in humans. It is worth noting that the analysed air filters contained, among others, *Escherichia coli*, which produces endotoxins with strong pro-inflammatory (immunotoxic) effects in humans. The presence of Gram-negative bacteria in air filters, is equivalent to the presence of endotoxins in the MBT workplace bioaerosol. However, it must be clearly emphasised that the results of the present study do not allow for determining endotoxin concentrations. Occupational exposure to organic dust containing high concentrations of endotoxins may cause inflammatory respiratory diseases such as hypersensitivity pneumonitis and organic dust toxic syndrome (ODTS). A significant association has also been demonstrated between endotoxin exposure and allergic asthma [30]. In the authors’ study were detected bacteria *E. coli*. Among this species the O157:H7 strain (however in the authors’ study was detected *E. coli* as species) is recognised by WHO as the most clinically significant serotype among Shiga toxin-producing *E. coli* from a public health perspective. Infected individuals develop gastrointestinal symptoms, including abdominal cramps and diarrhoea, which may progress to haemorrhagic colitis. The fatality rate ranges 3–5%. Reservoirs include livestock as well as domestic animals such as dogs and cats, and poultry. Transmission occurs mainly via ingestion of contaminated food. Humans may also become carriers, and person-to-person spread occurs via the faecal-oral route [32]. In MBT facilities, in addition to the sources mentioned above, human faecal material from used adult diapers originating from hospital patients may also serve as a source of pathogens. Therefore, it cannot be ruled out that occupational exposure combined with insufficient hygiene, may lead to infection or acquisition of carrier status among MBT facility workers.

Among the detected bacteria were present Actinomycetes, including among others *Bacillus, Thermobifida, Nocardiopsis* taxa that are capable of producing bioactive compounds. Enzymes can cause allergenic effects in individuals exposed via inhalation. In persons sensitised to proteins contained in these enzymatic molecules or genetically predisposed to allergies, inhalation exposure may trigger allergic symptoms affecting the respiratory system, nasal mucosa, and eyes [30].

The study results demonstrated also the presence of bacteria of the taxa *Streptomyces* on the air filters. These Gram-positive, spore-forming bacteria, belonging to the Actinomycetes, possess the ability to produce numerous biologically active compounds, including antibiotics, immunosuppressive agents, and hydrolytic enzymes. Certain species are used in the industrial production of immunosuppressive drugs intended to reduce immune system activity. Several *Streptomyces* species are involved in the development of pulmonary diseases, and some may directly cause hypersensitivity pneumonitis. At the current stage of scientific knowledge, it is difficult to precisely determine the health effects for exposed workers; exposure to these bacteria may exert both negative (e.g., immunosuppression) and potentially beneficial effects (e.g., reduction of inflammatory responses). The ultimate health outcome is determined by multiple factors, including the species composition of the bioaerosol, the duration and frequency of exposure (length of employment, shift system), as well as individual characteristics (e.g., genetics, microbiota composition, health status, existing diseases, age) [33].

Some bacterial species detected in the studied environment possess the ability to form biofilm – a multilayered, “living” matrix composed of densely interconnected bacterial cells, such as *Staphylococcus aureus* [29]. The capacity to form biofilms on devices and objects used within the work hall, as well as on personal items (e.g., mobile phones, jewellery), increases the risk of transmitting these bacteria via such objects to other areas of the work environment (e.g., break rooms, sanitary facilities, offices) and beyond the workplace (primarily to workers’ homes).

It is speculated that occupational exposure to the bacteria, especially opportunistic, that were also detected in this study may disrupt the species balance within the microbiome of the skin and respiratory tract, particularly the nose, oral cavity, pharynx, and intestines. The human microbiome is composed of multiple specialised microbial communities, including commensal species (not causing disease in humans), symbiotic species (beneficial to both host and microbe), and pathogenic species (disease-causing in humans). The species composition of these communities is closely linked to states of health and disease, as individual strains possess specific functional properties, such as the breakdown of dietary substrates, or the production of vitamins, neurotransmitters, hormones, and other bioactive compounds essential for host physiology. Disturbances in the species composition of the microbiome resulting in an overgrowth of pro-inflammatory bacteria at the expense of species beneficial to the host are referred to as dysbiosis. Dysbiosis of the intestinal microbiota has been associated with numerous diseases, including inflammatory bowel disease, metabolic disorders (e.g., type II diabetes, obesity), autoimmune diseases, cancer, and even depression. Some scientific literature indicates that the hypothesis about the negative influence of the occupational exposure to high concentrations of bioaerosols on workers’ microbiomes dysbiosis cannot be eliminated [29].

When assessing the qualitative aspects of occupational exposure to bacteria present in the working environment, it is necessary to take into account the applicable legal regulations as well as current scientific knowledge. In Poland, the legal basis is the Regulation of the Minister of Health of April 22, 2005 on harmful biological agents in the working environment and on the protection of the health of workers occupationally exposed to such agents. The classification and list of harmful biological agents is provided in Annex 1 to the amendment of this regulation, which entered into force in 2020 [24]. According to this legal act, among the bacteria with a relative abundance above the ≥0.5% threshold, only the species *Saccharomonospora viridis* is classified in hazard group 2. *Saccharomonospora viridis* is a thermophilic species belonging to the Actinomycetes. The literature indicates that these bacteria are detected in the working environment of waste composting plants. In humans, exposure to *S. viridis* has been associated with hypersensitivity pneumonitis caused by organic dust. Therefore, the risk of this disease should be taken into account in the occupational risk assessment for employees of MBT facilities [34]. Pathogenic bacteria may cause infections in exposed individuals, particularly those with reduced immunity, even when present at low concentrations. Analysis of the results, irrespective of relative abundance, revealed the presence of opportunistic and pathogenic bacteria, including species belonging to risk group 3 [24], which are capable of causing severe human diseases and may spread between people, although effective prophylaxis or treatment exists. Among these bacteria detected on the analysed filters were *Bacillus anthracis*, *Burkholderia pseudomallei*, *Mycobacterium leprae, Mycobacterium tuberculosis and Mycobacterium ulcerans*. With the exception of *M. ulcerans*, these bacteria may be transmitted via the respiratory route, which significantly increases the risk of infection among exposed workers. The presence of *B. anthracis* is highly likely to originate from soil, traces of which enter mixed municipal waste. However, the risk of anthrax among MBT facility workers, provided the basic hygiene practices are observed, should be considered negligible [35].

*Mycobacterium tuberculosis* detected in MBT facilities in the present study most likely also originates from faecal material of hospital patients [36]. However, the probability of MBT workers in Poland becoming infected with tuberculosis appears to be very low. Nevertheless, to fully exclude or confirm such a risk, studies using traditional microbiological methods are required to verify the viability of *M. tuberculosis* in the bioaerosol.

*Mycobacterium ulcerans* was present on as many as 8 out of the 9 analysed air filters. This species causes necrotising infections of the skin and soft tissues in humans (Buruli ulcers). Although classified as a tropical disease, scientific literature reports its spread to other parts of the world. The environmental transmission routes of this pathogen remain unknown; however, in Australia, possums have been identified as carriers, and in other regions it is suspected that mosquitoes and biting aquatic arthropods may act as vectors [37]. Currently, there are no scientific reports confirming the occurrence of *M. ulcerans* in Poland, making it difficult to draw conclusions about the sources of these bacteria in MBT facilities. Nonetheless, factors such as human migration from tropical regions, where carriers are present as well as global climate warming should be considered.

When analysing the pathogenicity of bacteria present in the working environment, it is also important to consider the ESKAPE group of bacteria, which, according to the One Health framework, constitute the most common cause of healthcare-associated infections worldwide [25]. In the analysed air filters, the presence of all pathogens from this group was confirmed: *Enterococcus faecium*, *Staphylococcus aureus*, *Klebsiella pneumoniae*, *Acinetobacter baumannii*, *Pseudomonas aeruginosa*, and *Enterobacter* spp. It is highly likely that these bacteria originate from hospital waste (used adult diapers from hospital patients), although an animal origin is also possible (dog faeces and remnants of raw animal-derived food). The risk of workers in MBT facilities acquiring carrier status through airborne and contact exposure may be considerable. Consequently, they may become vectors of these pathogens in their surroundings, particularly posing a risk to members of their households.

Another significant health threat to workers in MBT facilities is antimicrobial resistance, currently a major global public health challenge. From an individual perspective, it means that infections caused by antibiotic-resistant bacteria are more difficult and prolonged to treat, and may, in some cases, result in death [25]. Resistance genes can be transferred between different bacteria, both within the same species and between phylogenetically distant species. In the analysed air filters, genes encoding bacterial resistance to antibiotics commonly used in human medicine were detected. The WHO has published a list of medically important antibiotics as a risk-management tool to reduce antimicrobial resistance arising from their use outside human medicine [38]. Antibiotics are categorised into 3 main groups according to their authorised use: those authorised exclusively for humans; those used only in animals (not authorised for human medicine); and those authorised for use in both humans and animals.

In the analysed material, genes were detected that encode resistance to:

– antibiotics authorised exclusively for human use (carbapenems, glycopeptides, glycylcyclines, monobactams, oxazolidinones);
– antibiotics authorised for both humans and animals including critically important antimicrobials (CIA), such as aminoglycosides, macrolides (14-, 15- and 16-membered ring), and ansamycins (rifamycins);
– highly important antimicrobials (HIA), such as cephalosporins, cephamycins, lincosamides, penams/penicillins, streptogramins, sulphonamides, and tetracyclines;
– highest-priority critically important antimicrobials (HPCIA), such as fluoroquinolones;
– important antibiotics (IA), such as pleuromutilins.

Among drugs not authorised for human use, a gene conferring resistance to aminocoumarins was identified in the studied air filters. The presence of genes encoding multidrug resistance, which poses a particularly high health risk to exposed workers, should also be highlighted.

The most frequently detected resistance mechanisms included resistance to aminocoumarins (*parY*), glycopeptides (*vanJ*), lincosamides (*lnuA*), phenicols (*catA8*, *cml*, *cmx*), and sulfonamides (*sul1*, *sul2*), as well as multidrug resistance to lincosamides, macrolides, and streptogramins (*ermA*, *ermB*, *ermC*). Lincosamides and macrolides are effective against atypical bacteria [30]. The study also demonstrated the presence of genes encoding resistance to β-lactams (penicillins) and fluoroquinolones an important finding, as these antibiotic classes are effective in the initial treatment of severe infections and reduce mortality due to antimicrobial-resistant bacterial infections by >70% [39].

The frequency of resistance-gene detection in the examined air filters may be associated with the current epidemiological situation in the region, the density of the treated population, and socio-economic characteristics specific to that region [38]. However, there is important to note that the correlation between genotype and phenotype is not always consistent. The lack of phenotypic confirmation of antimicrobial resistance belongs to the limitations of the study and is explained in appropriate section.

Current scientific knowledge allows for drawing conclusions about the health effects caused by individual taxa of the bacteria detected isolated in this study. However, there is still insufficient understanding to enable an assessment of the impact of exposure to bioaerosols with a complex qualitative composition, such as those present in municipal waste processing facilities. the authors’ study expands global knowledge on the bacterial species present in the air of MBT installations and may serve as a basis for future epidemiological research on health outcomes among workers in these facilities, but it does not permit dose–response inference.

Filters from vehicles have also been used to assess occupational exposure to bioaerosol by other researchers. In Portugal, Viegas et al. [17] conducted a study, in which among other analyses were performed the high-throughput sequencing to identify bacterial and fungal taxa in organic dust from 17 air conditioning filters from forklifts at a single municipal waste sorting facility. However, because the aim of this study was to assess the toxicity of dust collected on air filters, only the dominant bacteria in the studied material (*Paracoccus marcusii, Leuconostoc mesenteroids*, and *Haererehalobacter salari*a) and those causing proinflammatory responses in humans (*Epicoccum nigrum, Brachybacterium conglomeratum, Staphylococcus sciuri, Psychrobacter sanguinis, Leuconostoc mesenteroides*, and *Myroides odoratimimus*) were described at the species level. Among these species, only *P. sanguinis*, *L. mesenteroides*, and *M. odoratimimus* were detected in the authors’ study, which were present on all tested air filters but 2–3 orders of magnitude below the ≥0.5% threshold. Portuguese researchers described the diversity of bacterial microflora on forklift filters at a more general level than the authors did, limiting the results to the dominant bacterial families. This approach stemmed from the different purpose of this study, which was to analyse the cytotoxicity of organic dust collected on forklift filters in a waste sorting plant [17].

Informal information obtained during communication with MBT facilities while attempting to secure their participation in this study suggests that OHS procedures that should govern the management of air filters supplying air to the cabins of utility vehicles in MBT installations are being violated. It appears that improper handling of air filters is driven by an intention to reduce expenses associated with purchasing new filters. Thus the findings of this study underscore the need to implement rigorous preventive measures in MBT facilities to protect the health of their employees and other individuals present on site, particularly delivery truck drivers. The study has already been applied in the form of detailed hygiene recommendations for workers performing tasks under conditions of exposure to bacteria and other biological agents in municipal waste processing and disposal facilities.

### Limitations

The primary limitation of the study is the small number of analysed filters and the lack of documented information regarding their duration of use. The available variables were insufficient to perform statistical analyses and to draw conclusions about factors influencing the biodiversity of the bacterial microflora present in the organic dust accumulated on the filtering material. Furthermore, the study was conducted in only 1 season, although the air filters had been used in MBT facilities during the summer months, when exposure to bacteria in waste-management facilities is at its annual peak.

The use of high-throughput sequencing does not allow for a quantitative assessment of exposure to bacterial bioaerosols. Moreover, it is not possible to determine which of the bacterial species identified by NGS were viable and capable of infecting humans. At the same time, there is no basis for assuming that the detected genetic material originated exclusively from non-viable bacterial cells. It should also be clearly emphasised that the percentage relative abundance used as a quantitative metric to facilitate interpretation of the results is not equivalent to the concentration of bacteria in workplace air. What is more, ARGs were detected exclusively by NGS, which does not confirm the phenotypic antimicrobial resistance of bacteria present in the MBT environment. The correlation between genotype and phenotype is not always consistent. The detection of resistance genes should be interpreted as an indicator of potential genetic resistance to antimicrobials, rather than as direct evidence of phenotypic resistance. In the present study, no direct phenotypic susceptibility testing was performed; therefore, it remains unknown whether the detected genes were actually expressed and associated with clinically relevant resistance.

The irregularities in the handling of air filters described in the Results section were obtained informally through telephone conversations and originated from most, but not all, facilities participating in the study. Additionally, information provided by other MBT facilities that declined to supply air filters for this study was considered. Therefore, these data should be treated solely as noteworthy observations and not as scientifically robust evidence comparable to findings derived from research conducted using standardised questionnaire-based or interview-based survey tools. the authors decided to publish these observations due to the concern they raise regarding inadequate attention to the health and safety of both, utility-vehicle drivers and other MBT plants workers.

### Data availability

The paired-end sequences of metagenomic samples collected in this study are publicly available in the National Center for Biotechnology Information under the BioProject ID PRJNA1373541 [40].

## CONCLUSIONS

Although the number of analysed air filters was limited, the genetic analysis provides a basis for the following conclusions:

1. The study demonstrated a high species diversity of environmental, human-associated, and animal-associated bacteria present on air filters in MBT facilities.
2. Workers in MBT facilities are exposed to bacteria with high pathogenic potential, including opportunistic and pathogenic taxa species or strains classified into risk groups 2 and 3.
3. ESKAPE bacteria were present in the working environment of MBT facilities.
4. The study confirmed the presence of genes encoding resistance to antibiotics authorised exclusively for human use, those authorised for both humans and animals, and those not authorised for human use. Genes encoding resistance to single antibiotic classes as well as genes conferring multidrug resistance were detected.
5. Air filters from utility vehicles used in MBT installations can be used to the identification of bacteria present in the working environment; however, the lack of documented information on filter usage conditions in the workplace prevents analysis of the relationship between the results obtained and workplace conditions.
6. The findings may serve as a basis for incorporating bacteria isolated from air filters into occupational risk assessments for workers in MBT facilities in Poland and other European countries.
7. There is a need to verify the effectiveness of control measures for MBT facility workers and drivers delivering waste to these facilities with regard to preventing the acquisition of infections or carrier status during occupational activities.
8. The study provides a foundation for formulating new research hypotheses concerning the environmental transmission pathways of selected bacterial pathogens.

## AI USE

The authors declare that an AI was used to improve the language, while all intellectual work, including developing the research concept, reviewing the literature, developing the methodology, collecting the material, performing the results analysis and interpretation, drawing conclusions, and writing the manuscript text, was carried out by the authors.
